# The molecular and structural bases for the association of complement C3 mutations with atypical hemolytic uremic syndrome

**DOI:** 10.1016/j.molimm.2015.03.248

**Published:** 2015-08

**Authors:** Rubén Martínez-Barricarte, Meike Heurich, Andrés López-Perrote, Agustin Tortajada, Sheila Pinto, Margarita López-Trascasa, Pilar Sánchez-Corral, B. Paul Morgan, Oscar Llorca, Claire L. Harris, Santiago Rodríguez de Córdoba

**Affiliations:** a Centro Investigaciones Biológicas, Ramiro de Maeztu 9, 28040 Madrid, Spain; b Ciber de Enfermedades Raras, Ramiro de Maeztu 9, 28040 Madrid, Spain; c Institute of Infection & Immunity, School of Medicine, Cardiff University Heath Park, Cardiff CF14 4XN, United Kingdom; d Unidad de Inmunología, Hospital Universitario La Paz-IdiPAZ, and Ciber de Enfermedades Raras. Paseo de la Castellana 261, 28046 Madrid, Spain; e Unidad de Investigación, Hospital Universitario La Paz-IdiPAZ, and Ciber de Enfermedades Raras. Paseo de la Castellana 261, 28046 Madrid, Spain

**Keywords:** AP, alternative pathway, aHUS, atypical hemolytic uremic syndrome, CP, classical pathway, CR1, complement receptor 1, DAF, decay accelerating factor, DDD, dense deposit disease, EM, electron microscopy, FB, factor B, FD, factor D, FH, factor H, FI, factor I, KD, equilibrium dissociation constant, LP, lectin pathway, MAC, membrane attack complex, MCP, membrane cofactor protein, mt, mutant, sMCP, soluble recombinant membrane cofactor protein (MCP, CD46), SCR, short consensus repeat, SPR, surface plasmon resonance, wt, wild-type, Complement C3, C3 mutation, MCP, Factor H, TED, Atypical hemolytic uremic syndrome

## Abstract

Atypical hemolytic uremic syndrome (aHUS) associates with complement dysregulation caused by mutations and polymorphisms in complement activators and regulators. However, the reasons why some mutations in complement proteins predispose to aHUS are poorly understood. Here, we have investigated the functional consequences of three aHUS-associated mutations in C3, R592W, R161W and I1157T. First, we provide evidence that penetrance and disease severity for these mutations is modulated by inheritance of documented “risk” haplotypes as has been observed with mutations in other complement genes. Next, we show that all three mutations markedly reduce the efficiency of factor I-mediated C3b cleavage when catalyzed by membrane cofactor protein (MCP), but not when catalyzed by factor H. Biacore analysis showed that each mutant C3b bound sMCP (recombinant soluble MCP; CD46) at reduced affinity, providing a molecular basis for its reduced cofactor activity. Lastly, we show by electron microscopy structural analysis a displacement of the TED domain from the MG ring in C3b in two of the C3 mutants that explains these defects in regulation. As a whole our data suggest that aHUS-associated mutations in C3 selectively affect regulation of complement on surfaces and provide a structural framework to predict the functional consequences of the C3 genetic variants found in patients.

## Highlights

• Mutations in C3 have been associated with aHUS and other glomerulopathies.
• aHUS-associated C3 mutants R592W, R161W, and I1157T impair regulation by MCP, but not by FH.
• EM analysis provides the structural basis for the functional impairment of the R161W and I1157T mutants.
• Data supports aHUS-associated C3 mutations selectively affect complement regulation on surfaces.

## 1 Introduction

Hemolytic uremic syndrome (HUS) is a severe renal disorder characterized by Coomb's negative microangiopathic hemolytic anemia, thrombocytopenia and acute renal failure. Typical HUS usually follows a diarrheal episode associated with infection by Shiga toxin producing 0157:h7 *Escherichia coli*. Between 5 and 10% of HUS cases are not associated with infection; these have a poor prognosis with multiple recurrences and often progress to acute renal failure, with a 30% mortality rate, reduced significantly since the introduction of anti-complement therapeutic Eculizumab. The majority of cases of this atypical form of HUS (aHUS) are associated with mutations or polymorphisms in complement proteins. Renal injury in aHUS is caused by complement-mediated endothelial damage with formation of microthrombi that occlude kidney arterioles. Arteriole occlusion causes one of the hallmarks of HUS, the presence of schistocytes, fragments produced as erythrocytes break up when passing at high pressure through partially occluded vessels.

Complement is a major component of innate immunity with crucial roles in microbial killing, apoptotic cell clearance, immune complex handling and modulation of adaptive immune responses. Complement activation by three independent activation pathways, classical (CP), lectin (LP) and alternative (AP), results in formation of meta-stable protease complexes, C3-convertases (AP, C3bBb; CP/LP, C4b2a), that cleave C3 to generate C3b. Convertase-generated C3b forms more AP C3-convertase, providing exponential amplification of initial activation. Binding of C3b to the C3-convertase generates a C5-cleaving enzyme (C5-convertase) that initiates formation of the lytic membrane attack complex (MAC). In health, activation of C3 is restricted and deposition of C3b and further activation of complement limited to the surface of pathogens by multiple regulatory proteins, including factor H (Sansbury et al., 2014), C4b-binding protein (C4BP), membrane cofactor protein (MCP), decay accelerating factor (DAF), complement receptor 1 (CR1) and CD59, that control complement activation and prevent consumption of components by inactivating C3b or C4b (factor I-cofactor activity), dissociating the C3/C5 convertases (decay accelerating activity), or inhibiting MAC formation (Walport, 2001a, Walport, 2001b).

Mutations in genes encoding the regulatory proteins FH (*CFH*), MCP (*MCP*) and FI (*CFI*), and the complement activating components factor B (*CFB*) and C3, are associated with aHUS; importantly, aHUS-associated mutations in regulators are loss-of-function, while mutations in activators are gain-of-function (Richards et al., 2001, Richards et al., 2003, Pérez-Caballero et al., 2001, Goicoechea de Jorge et al., 2007, Caprioli et al., 2001, Caprioli et al., 2003, Esparza-Gordillo et al., 2005, Sartz et al., 2012, Roumenina et al., 2012, Noris et al., 2003, Sánchez-Corral et al., 2002, Warwicker et al., 1998, Frémeaux-Bacchi et al., 2008). We present here the functional and structural characterization of three C3 mutations found in the Spanish aHUS cohort. All three mainly impair regulation of the AP convertase on surfaces by MCP (CD46), an effect consistent with the pathogenic mechanisms that characterize aHUS, and we provide a structural basis for this impairment. Further, we show that concurrence of these C3 mutations with aHUS-conferring risk polymorphisms in the *CFH* and *MCP* genes modulates penetrance and clinical severity of disease in aHUS.

## 2 Patients, materials and methods

### 2.1 Patients

Our series of aHUS patients comprises 237 unrelated individuals, including 214 Spaniards, seven from other European countries, six from the USA, six from South America and four from Tunisia. aHUS was diagnosed by the presence of one or more episodes of microangiopathic hemolytic anemia and thrombocytopenia defined on the basis of hematocrit (Ht) < 30%, hemoglobin <10 mg/dl, serum lactate dehydrogenase (LDH) > 460 U/L, undetectable haptoglobin, fragmented erythrocytes in the peripheral blood smear, and platelet count <150,000/μl, associated with acute renal failure. Patients with Stx-HUS, defined as the presence of Shiga toxin in the stools (by the Vero cell assay) and/or of serum antibodies against Shiga toxin (by ELISA) and/or LPS (O157, O26, O103, O111 and O145, by ELISA) were excluded. ADAMTS13 functional levels were used to exclude thrombotic thrombocytopenic purpura.

### 2.2 Clinical data from the families and the sporadic patient carrying C3 mutations

#### 2.2.1 Family HUS19

There are three affected individuals (HUS19, II-1, HUS19M, I-1 and HUS19T, I-2) in this Spanish pedigree, all of them alive. Patient HUS19 is a 12y-old female who first presented at the age of 14 months after a respiratory infection. She has had 8 recurrences thus far. In all these occasions she was treated with peritoneal dialysis, plasmapheresis and plasma infusion and recovered renal function. The family history revealed that the mother (HUS19M) and a maternal aunt (HUS19T) also suffered an episode of aHUS of unknown origin without recurrence. The mother presented with aHUS when she was 6 years old and recovered completely her renal function. The aunt had developed aHUS of unknown origin when she was 14 months and also recovered her renal function. However, some neurological deficits remained (epilepsy secondary to hypertensive crisis) in this patient.

#### 2.2.2 Family HUS107

Patient HUS107 is a female who belongs to another Spanish pedigree. Her mother was also affected. Patient HUS107 has a healthy daughter and two unaffected brothers; one of them also has two healthy daughters. HUS107 presented with aHUS at the age of 35 years associated with the intake of oral contraceptives. This episode was complicated by neurological disorders and was treated with antibiotics, immunosuppressive drugs (vincristine), plasmapheresis and plasma infusions. Currently, she has chronic renal insufficiency and is supported on peritoneal dialysis. Her mother presented at the age of 23 and also suffered aHUS associated with neurological complications. She received a cadaveric kidney graft at the age of 28 and died at the age of 31 with no evidence of recurrence in the grafted kidney.

#### 2.2.3 Patient HUS193

This patient presented with aHUS without a clear triggering factor. The biopsy showed thrombotic microangiopathy and he recovered completely after 8 months with haemodialysis. However, 16 years later he developed terminal renal disease. He is currently on haemodialysis waiting for a transplant. This patient shows permanent hypocomplementemia with low levels of C3 and CH50 but normal levels of C4.

### 2.3 Mutation screening/genotyping

The patients were screened for mutations and polymorphisms in *CFH*, *MCP*, *CFI*, *THBD*, *CFB* and *C3* genes by automatic DNA sequencing of PCR amplified fragments. Genomic DNA was prepared from peripheral blood cells according to standard procedures (Miller et al., 1988). Each exon of those genes was amplified from genomic DNA by using specific primers derived from the 5′ and 3′ intronic sequences as described (Richards et al., 2003, Pérez-Caballero et al., 2001, Fremeaux-Bacchi et al., 2004, Miller et al., 1988, Delvaeye et al., 2009). Automatic sequencing was performed in an ABI 3730 sequencer using a dye terminator cycle sequencing kit (Applied Biosystems, Foster City, CA). Copy number variations in the *CFHR1-R3* genes were analyzed by MLPA as described elsewhere (Abarrategui-Garrido et al., 2009).

### 2.4 Purification of complement components and activation fragments

FH and factor B (FB) were purified from plasma of healthy donors by a two-step method as described before (Tortajada et al., 2009). Briefly, filtered plasma was applied to a 5 ml affinity column to which 10 mg of mouse anti-human FHmAb (35H9) or anti-human FBmAb (D2) was coupled. Bound protein was eluted and polished by gel filtration on a SuperoseTM 6 10/300 column (GE Healthcare, Chalfont St. Giles, UK). sMCP (standing for soluble MCP) is a recombinant variant of MCP (also known as CD46) containing the first four SRCs (SCR1-4), lacking the membrane GPI anchor. sMCP was a generous gift from our collaborator Prof. Susan Lea, Oxford University. sMCP has been previously used to determine binding affinity with immobilized C3b (Heurich et al., 2011). All mutant and native C3 were purified from C3102R homozygotes as described before (Martínez-Barricarte et al., 2010). Briefly, C3 was prepared from plasma containing 20 mM EDTA and 10 mM benzamidine. Supernatant of a 10% (w/v) sodium sulphate precipitation was applied to a lysine-sepharose column. The flow through was collected and applied to a DEAE-Sepharose anion exchange column. Protein was fractionated using a NaCl gradient and C3-containing fractions were identified by ELISA and applied to a Mono S HR 5/5 cation exchange column (GE Healthcare). Protein was eluted and C3-containing peak fractions were pooled and preserved frozen at −70 °C. C3b was generated by limited digestion with trypsin as previously described (Sánchez-Corral et al., 1989). All C3b preparations were polished by gel filtration on a Superose 6 10/300 column (GE Healthcare) before being used to eliminate any detectable contaminants or aggregates. The protocols used were identical for patient-derived mutant C3 and wt C3; attempts to separate mutant from wt C3 in patient preparations by differential elution from ion exchange matrices were unsuccessful.

### 2.5 Activation of C3

Purified C3 (500 μg/ml), FB (50 μg/ml) and FD (4 μg/ml) in 20 mM sodium phosphate buffer pH 7, 100 mM NaCl and 2 mM MgCl_2_ were incubated in a water bath at 37 °C. Aliquots of 5 μl were extracted from the mix at 0, 0.5, 1, 2, 4, 8, 16, 32 and 60 min, mixed with SDS-PAGE sample buffer (2% SDS, 62.5 mM Tris, 10% glycerol and 0.75% bromophenol blue) to stop the reaction and loaded into a 10% reducing SDS-PAGE gel. The gels were stained using Coomassie brilliant blue R-250 (Biorad) and digitized using a GS-800 densitometer (BioRad) and the MultiGauge software package (FUJIFILM).

### 2.6 Factor H and sMCP cofactor activity for FI-mediated proteolysis of C3b

The cofactor activities of FH and recombinant sMCP were determined in a C3b proteolytic assay using purified proteins. In brief, C3b, FH or sMCP and FI were mixed in 10 mM Hepes pH 7.5, 150 mM NaCl, 0.02% Tween 20. Final concentrations in the first set were 350 μg/ml (C3b), 17.5 μg/ml (FI) and 13 μg/ml (sMCP) and in the second set they were 76 μg/ml (C3b), 3.8 μg/ml (FI). Mixtures were incubated at 37 °C in a water bath and aliquots containing 2 μg of C3b collected at 0, 1, 5, and 10 min. The reaction was stopped by dilution in 5 volumes of SDS sample buffer. Samples were analyzed in 10% SDS-PAGE under reducing conditions. Gels were stained with Coomassie brilliant blue R-250 (BioRad) and proteolysis of C3b determined by analyzing the cleavage of the α′-chain normalizing with the beta chain after scanning the gels with a GS-800 calibrated densitometer (BioRad) and the MultiGauge software package (FUJIFILM).

### 2.7 Surface plasmon resonance

Binding affinities of the disease-associated C3 mutants with regulators were measured by surface plasmon resonance (SRP) using a Biacore T100 instrument (GE Healthcare). A Biacore series *S*-carboxymethylated dextran (CM5) sensor chip (GE Healthcare) was prepared by immobilizing 400 RU C3b (Complement Technology) using standard amine coupling. The reference surface of the chip was prepared by activating and blocking the chip surface. Experiments were performed at 25 °C and at 20 μl/min. Concentration series of proteins were injected as triplicates in 10 mM HEPES-buffered saline with 3 mM EDTA and 0.05% (v/v) surfactant p20 (HBSEP; GE Healthcare). Factor H (0.07 μM to 4.18 μM) was flowed across C3b for 180 s to achieve steady-state conditions, followed by 300 s dissociation in running buffer. The surface was regenerated between analyte injections using 1 M NaCl, 0.1 M sodium acetate, pH 4. For MCP affinity analysis, the surface was adjusted to 1500RU C3b by thioester deposition, achieved by flowing FB and FD in the presence of Mg^2+^ to form C3 convertase followed by mutant C3 (or wt C3) as substrate (Harris et al., 2007). Varying concentrations of MCP (0.078 μM to 5 μM) were flowed over the C3b surface for 90 s to achieve steady-state, followed by dissociation using running buffer for 300 s. Data were processed using the BIAevaluation software (GE Healthcare). Data were double-referenced by subtracting RU shifts from the reference cell and a blank (buffer) injection; dissociation constants were calculated using steady-state affinity analysis.

MCP-cofactor activity was assessed on the mutant C3b surface and compared directly to the wt C3b surface. Mutant and wt C3b were deposited on the chip via the thioester adjusted to an equal density of 1500RU. The C3 convertase was formed prior to FI cleavage (0 s) by flowing FB (2.38 μM) and FD (42 nM) in the presence of Mg^2+^ over each surface separately; this confirmed identical convertase formation on both C3b surfaces. A fixed concentration of MCP (0.1 μM) and FI (57 nM) was then flowed across the C3b-coated surface at 10 μl/min using four sequential injections (for 60 s, 60 s, 90 s and finally for 120 s) cleaving C3b to iC3b, resulting in subsequent lack of affinity for FB. C3 convertase formation was measured at time zero and after each injection (% of initial convertase at time 0 s equals 100%) and plotted against time of MCP/FI exposure to the C3b surface for both mutant and wt C3b. GraphPad Prism Version 5.01 software was used to analyze linear regression of C3 convertase formation (RU) versus log time (s) yielding the slope and the goodness-of-fit (*r*^2^) for each curve. The software compared the slopes by calculating a *P* value (unpaired two-tailed) that determines the differences in slope values for the mutant versus the wt C3b surfaces, determining whether the lines are significantly different. The inter-experimental variation was determined by comparing the slopes for separate wt C3b experiments against each other and did not differ significantly.

### 2.8 Electron microscopy and image processing of C3b mutants

C3b, C3b_R592W_, C3b_R161W_ and C3b_I1157T_ were adsorbed on carbon-coated grids and stained using 2% uranylformate. All observations were performed using a JEOL-1230 at 100 kV and images of mutant and wt C3b were recorded using a TVIPS F416 CMOS camera and a final magnification of 54926. Images of individual molecules for all samples (6712, 7964, 7327 and 6539 images for C3b, C3b_R592W_, C3b_R161W_ and C3b_I1157T_, respectively) were extracted automatically and subsequently classified and averaged using XMIPP (Sorzano et al., 2004). Image processing and averaging revealed that most views of the native and mutant C3b molecules could be assigned to views of C3b in which the TED domain was clearly either attached or displaced from the MG ring. However, there were some images in all C3b samples that could not be assigned to a specific conformation since they corresponded to views of the molecule where the position of the TED domain in reference to the MG ring could not be determined. These images corresponded to approximately 14% of the images in all experiments (1008, 1379, 1047 and 1520 images for C3b, C3b_R592W_, C3b_R161W_ and C3b_I1157T_ respectively), and they were discarded from the analysis.

## 3 Results

### 3.1 Identification of C3 mutations in aHUS patients

**Fig. 1:**
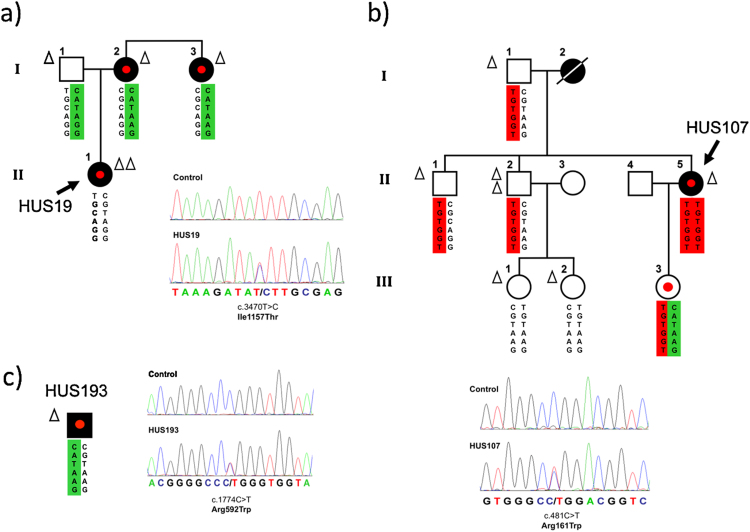
Mutations in the *C3* gene in three Spanish aHUS patients. Pedigrees of families (a) HUS19 and (b) HUS107, as well as patient (c) HUS193 are described. Individuals are identified by numbers within each generation (in Roman numbers). Affected individuals are indicated with solid symbols. The six SNPs that comprise the CFH haplotypes are represented in columns. The risk-associated CFH-H3 haplotype is squared in red. The protective CFH-H2 haplotype is squared in green. Each triangle indicates one copy of the MCP*_GGAAC_* risk haplotype. Patients are indicated by an arrow with the appropriate code. Carriers of the heterozygous C3 mutations are indicated with a red dot. For each *C3* mutation, the chromatogram corresponding to the DNA sequence surrounding the mutated nucleotide in *C3* is shown for the appropriate aHUS patient and for a control sample. The corresponding amino acid sequences for the wild type and the mutated alleles are shown. The amino acid and nucleotide numbering is referred to the translation start site (Met +1). (For interpretation of the references to color in this figure legend, the reader is referred to the web version of this article.)

The Spanish aHUS cohort comprises 237 unrelated individuals, including 214 Spaniards, seven other European, six USA, six South America, four Tunisia. Three patients (HUS19, HUS107, and HUS193; all Caucasians, Spanish origin; 1.3% of the cohort) carried miss-sense mutations in heterozygosis in the *C3* gene (Fig. 1). The mutation in HUS19 located in exon 27 (c.3470T > C) causes the amino acid substitution I1157T in the TED domain of C3. This mutation is present in the three aHUS-affected members in the HUS19 pedigree, all presenting normal C3 levels (Fig. 1a; Table 1). HUS107 carries a mutation in exon 4 (c.481C > T) which causes the amino acid substitution R161W in the MG2 domain of C3 and also associates with normal C3 levels. The mutation was found in the patient and her asymptomatic 10-year-old daughter (Fig. 1b). HUS107 likely inherited the mutation from her aHUS-affected mother, but no DNA was available to confirm this. HUS193 carries a mutation in exon 14, c.1774C > T, that causes the amino acid substitution R592W in the C3 MG6 domain (Fig. 1c); no family data were available. HUS193 shows low levels of plasma C3. The R161W, R592W and l1157T mutations have been previously associated with aHUS (Roumenina et al., 2012, Frémeaux-Bacchi et al., 2008, Schramm et al., 2015). Two of these gain-of-function C3 mutations, R161W and I1157T, are relatively prevalent mutations representing around 50% of the aHUS patients carrying C3 mutations in Europe and Japan, respectively (Schramm et al., 2015, Noris et al., 2010, Matsumoto et al., 2014, Maga et al., 2010, Fan et al., 2013). Importantly, these mutations are always found associated with aHUS. The objective of our work was to confirm previous functional characterization using recombinant proteins with plasma purified preparations of these prevalent and aHUS-specific mutant proteins, and to extend their functional characterization with structural data. None of the three C3 mutations described in this report were found in controls, including 128 normal Spaniards, or in public databases, including dbSNP, the 1000 genome database (1092 subjects, www.1000genomes.org) and the NHLBI GO exome sequencing projects (6503 subjects, evs.gs.washington.edu/EVS/) (October 2014). All three patients were C3S (C3102R) homozygotes.

The heterogeneity of the kidney outcome in our patients is consistent with that reported for other aHUS patients carrying the same mutations. In fact, from the 12 patients carrying the I1157T mutations with reported clinical descriptions, four developed a severe disease reaching end stage renal disease (ESRD), while 8 had a relapsing disease and/or achieved remission. The same was observed for patients carrying the R161W mutation; nine out of 14 reached ESRD and the remaining 5 had chronic renal disease (Roumenina et al., 2012, Noris et al., 2010, Matsumoto et al., 2014, Fan et al., 2013, Fremeaux-Bacchi et al., 2013).

### 3.2 aHUS risk polymorphisms in aHUS patients with mutations in C3

To investigate whether clinical variability of disease in *C3* mutation carriers is influenced by other previously reported *CFH* and *MCP* aHUS-associated polymorphisms, all patients and relatives were genotyped (Fig. 1; Table 1). Patient HUS19, who presented a more severe clinical phenotype than her affected mother and aunt, including earlier onset and multiple recurrences, carried the *MCP*_*GGAAC*_ risk haplotype in homozygosis, whereas her relatives carried a single copy of this haplotype and one copy of the aHUS-protective *CFH*_*GATAAG*_ haplotype (Fig. 1a). Patient HUS107 carried the *MCP*_*GGAAC*_ risk haplotype in heterozygosis and the *CFH*_*TGTGGT*_ risk haplotype in homozygosis. Her daughter (individual III-3 in pedigree HUS107; Fig. 1b), currently healthy, carried the same C3 mutation with both the protective *CFH*_*GATAAG*_ haplotype and the *CFH*_*TGTGGT*_ risk haplotype in heterozygosis. No family members were available for patient HUS193 who had a slowly developing disease and carried both the *MCP*_*GGAAC*_ risk haplotype and the *CFH*_*CATAAG*_ protective haplotype in heterozygosis (Fig. 1c).

### 3.3 Purification of C3 mutant proteins

C3 proteins were purified from plasma of appropriate mutant carriers; these individuals were heterozygotes and therefore C3 preparations were a mix of mutant and wild-type (wt) C3. Proteomic analysis of C3 proteins purified from patients confirmed the presence of mutant C3 (Supplementary Table 1); SDS-PAGE under reducing conditions separated the C3b β-chains corresponding to wt and mutant alleles in C3 from patients carrying the R592W and R161W mutations enabling us to estimate that mutant C3 protein represented approximately 50% of total circulating C3 in these patients (Supplementary Fig. 1). By using a mixture of wt and mutant protein preparation for our analyses, rather than making homogenous recombinant proteins, we provide a clearer picture of the in vivo functional effect as it presents in the patients.

### 3.4 aHUS-associated C3 mutants are activated normally by the AP C3 convertase

**Fig. 2:**
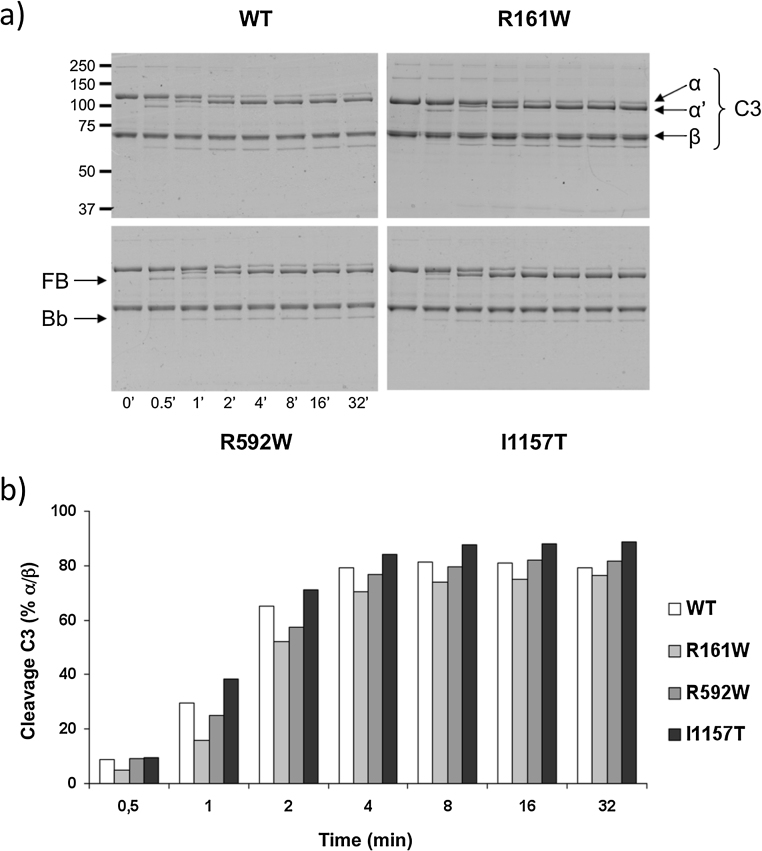
Activation assay of mutant C3. C3 was mixed with FB and FD and the cleavage of the C3 alpha chain to alpha′ as well as the cleavage of FB into Bb (indicated by arrows) was followed over time (time 0′ shows C3 control only), as shown (a) in SDS-PAGE gels for wt C3b (top left) and each heterozygous mutant C3b_R161W_ (top right), C3b_R592W_ (bottom left), and C3b_I1157T_ (bottom right). It is apparent from the gels (a) and the densitometry analysis (b) illustrating the C3 cleavage as % alpha/beta chain ratio, that all mutants and the wild type C3 are completely activated to C3b after 10 min of reaction, and confirmed by Bb generation. The experiment was repeated twice with identical results.

The three C3 mutant proteins were incubated with FB and FD at 37 °C and cleavage to C3b analyzed by SDS-PAGE. For comparison, wt C3 purified from pooled normal plasma at the same time and using the same method was included. Coomassie-stained gels and densitometry analyses are shown in Fig. 2. All three aHUS-associated C3 mutants were activated to C3b by the AP C3 convertase normally and at similar rates to wt C3.

### 3.5 Cofactor analysis of aHUS-associated C3 mutants reveals resistance to regulation by MCP but not FH

**Fig. 3:**
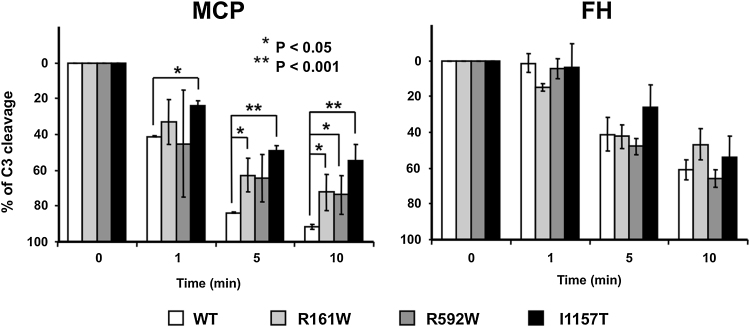
Cofactor activity of sMCP and FH for mutant C3b. (a) Recombinant sMCP cofactor activity assay shows that all heterozygous C3b mutants are more resistant than wt C3b to inactivation by FI in the presence of sMCP. After 10 min, only half of the mutant I1157T was cleaved. (b) FH cofactor activity assay shows that mutant and wt C3b are inactivated by FI in the presence of FH at similar rates and to similar degrees.

C3 preparations from each of the three individuals, and normal C3, were converted to C3b using trypsin (Sánchez-Corral et al., 1989) and used to test cofactor activities of FH and MCP for FI-mediated C3b cleavage. Identical amounts of each C3b preparation were incubated with FI and either purified FH or soluble recombinant MCP (sMCP). All three aHUS mutant C3b proteins were inactivated by FI in the presence of FH at a rate indistinguishable from that of wt C3b (Fig. 3b). In contrast, all three aHUS mutant C3b preparations were partially inactivated by sMCP (Fig. 3a). An average of 50% of C3b_I1157T_ and 70% of C3b_R592W_ and C3b_R161W_ was cleaved, compared to almost complete cleavage of wt C3 after 10 min. Considering that approximately 50% of the C3 in preparations from patients carrying the R592W and R161W mutations is mutant protein, these assays demonstrate significant resistance of the C3b_R592W_ and C3b_R161W_ mutant proteins to inactivation by MCP, almost complete in the case of the C3b_I1157T_ mutant. These data suggest that defective surface regulation is the primary consequence of these aHUS-associated C3 mutations.

### 3.6 C3b mutants bind MCP and FH with lower affinity compared to wt

**Fig. 4:**
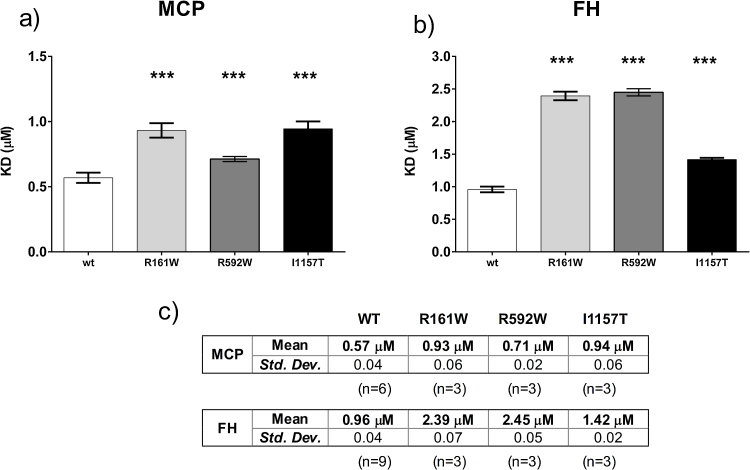
Affinity assays of MCP and FH for the mutant C3 mutants. Binding affinities of C3b mutants with complement regulators MCP and FH were measured by surface plasmon resonance (SRP). (a) Recombinant sMCP binding affinities (KD, μM) with C3b_R592W_ (0.71 ± 0.02 μM; *P* = 0.0006, *n* = 3), C3b_R161W_ (0.93 ± 0.06; *P* < 0.0001, *n* = 3), and C3b_I1157T_ (0.94 ± 0.06 μM; *P* < 0.0001, *n* = 3); all showing weaker affinity compared to wt C3b (KD 0.57 ± 0.04 μM, *n* = 6). (b) FH binding affinities (KD, μM) with C3b_R592W_ (2.45 ± 0.05 μM; *P* < 0.0001, *n* = 3), C3b_R161W_ (2.39 ± 0.07; *P* < 0.0001, *n* = 3), and C3b_I1157T_ (1.42 ± 0.02 μM; *P* < 0.0001, *n* = 3); all showing weaker affinity compared to wt C3b (KD 0.96 ± 0.04 μM, *n* = 9). *P*-values (two-tailed, unpaired *t*-test; depicted as asterisk) refer to the comparison of each mutant KD versus wt KD. (c) Table summarizing affinity (KD, μM; Mean ± standard deviation) values of recombinant sMCP or FH binding to immobilized wt and mt C3b.

**Fig. 5:**
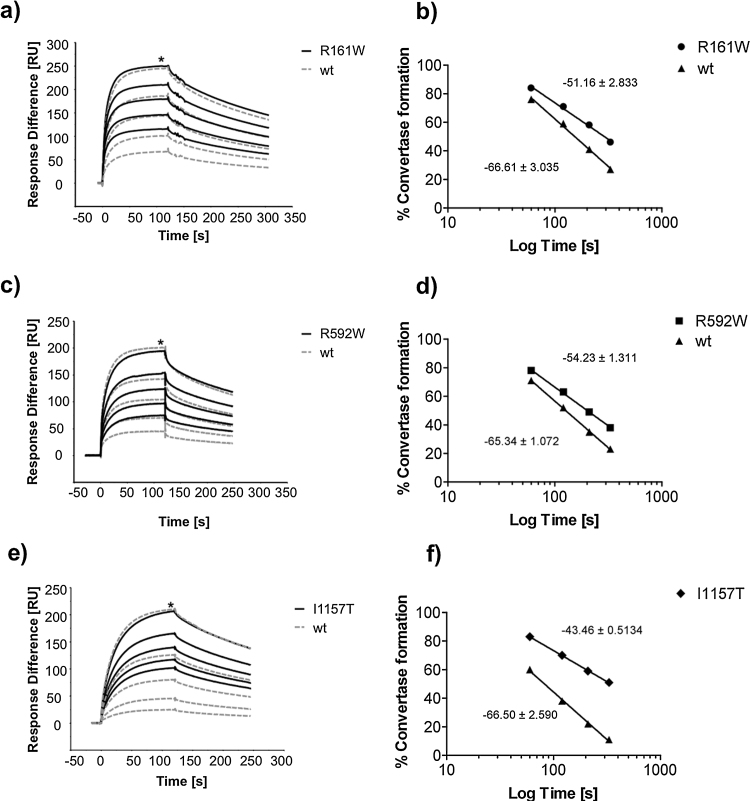
MCP cofactor assays for the C3 mutants. Sensorgrams show C3 convertase formation before (0 s) and after sequential (+60 s, 60 s, 90 s, 120 s) inactivation of C3b by sMCP and CFI. At time 0 s, formation of both mutant and wt C3 convertases are identical. C3 convertase formation (RU, maximum indicated by asterisk) remaining after sequential exposure to sMCP/FI relative to initial convertase (time zero) is shown for (a) C3b_R161W_, (b) C3b_R592W_, and (c) C3b_I1157T_ (solid black line) compared to wt C3b (dashed gray line). Linear graph (b, d, f) showing percentage C3 convertase formation vs log total exposure time (s) after each interval of C3b surface exposure to sMCP/FI. Linear regression analysis gives the slope for each curve, which illustrates the rate of C3b inactivation over time. Mutant (b) C3b_R161W_ (slope: −51.2 ± 2.85, *r*^2^ = 0.9939, *P* = 0.02), (d) C3b_R592W_ (slope: −54.2 ± 1.3, *r*^2^ = 0.9988, *P* = 0.003) and (f) C3b_I1157T_ (slope: −43.5 ± 0.5, *r*^2^ = 0.9997, *P* = 0.001) is resistant to sMCP/FI-mediated inactivation compared to wt C3b (slope: −66.5 ± 2.6, *r*^2^ = 0.9970). Individual wt C3b slopes were compared and showed no significant difference (*n* = 3, *P* = 0.91).

To determine whether mutant C3b binds MCP, each C3b preparation was immobilized as described in methods and sMCP flowed over the surface. The affinity of mutants C3b_R592W_, C3b_R161W_ and C3b_I1157T_ was reduced compared with wt C3b (Fig. 4a). In complementary experiments testing capacity of MCP to act as a cofactor in the FI-mediated inactivation of surface-bound C3b (Fig. 5a–f), we found that this decreased binding impaired MCP cofactor activity. MCP-cofactor activity was most impaired for C3b_I1157T_, directly corresponding to the cofactor activity results (Fig. 3a).

The affinity of all mutant C3b preparations for FH was reduced compared with wt C3b (Fig. 4b) but FH/FI cleavage assays showed no significant difference in FH-cofactor activity for C3b mutants compared to wt (Fig. 3b) in accordance with previous reports (Roumenina et al., 2012, Frémeaux-Bacchi et al., 2008).

### 3.7 Structural analysis of the C3b mutants

**Fig. 6:**
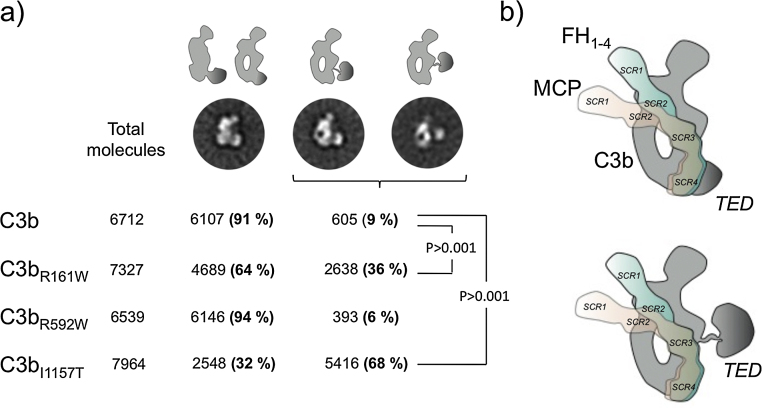
EM analyses of the overall structure of C3b, C3b_R592W_, C3b_R161W_ and C3b_I1157T_. (a) Galleries of three representative average images of wt C3b and heterozygous mutant C3b_I1157T_, C3b_R161W_ and C3b_R592W_ obtained by EM at 50 mM NaCl. The total number of molecules analyzed and the percentage of single molecule images assigned to each conformation after image processing is indicated, together with the statistic significance of their differences. A cartoon model for each of the conformations based on the structure of C3b is shown (TED domain highlighted in dark gray color). (b) Cartoon model of the interaction of the SCRs 1–4 of FH (FH1-4; green color) and SCRs 1–4 of MCP (rose color) with C3b (gray color) with the regular structure in the upper panel and with the one with the delocalized TED domain on the lower one, based on the crystal structure of C3b-FH (Wu et al., 2009) and the proposed model for the interaction between C3b and MCP (see also Fig. 7). (For interpretation of the references to color in this figure legend, the reader is referred to the web version of this article.)

**Fig. 7:**
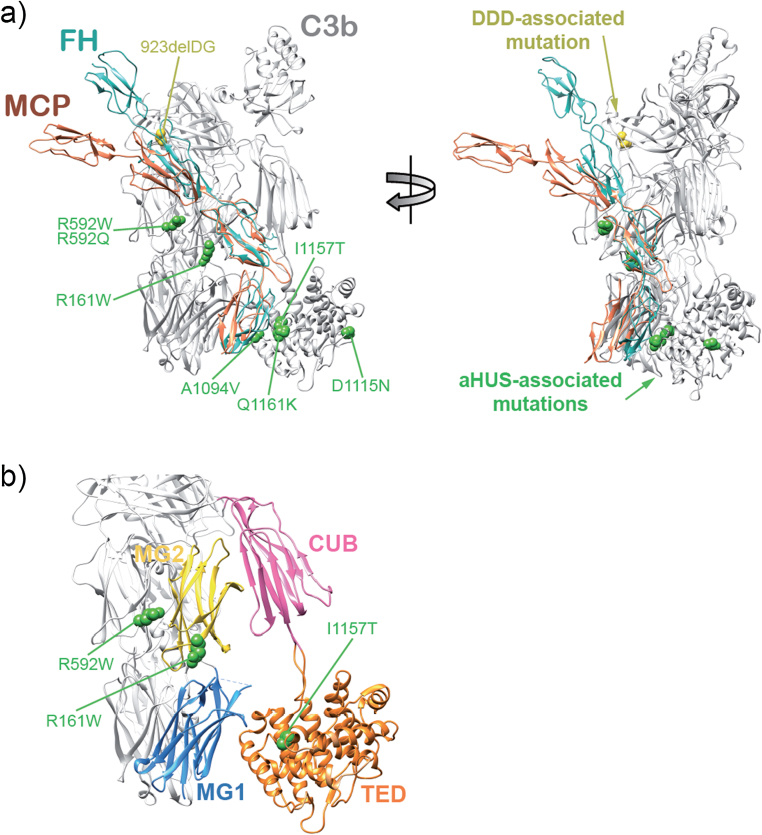
Structural implications for the aHUS-associated *C3* mutations. (a) Cartoon diagram (two views) of C3b in complex with the four SCRs of FH (PDB 2WII) and with MCP (3O8E) aligned at SCR3 with respect to FH. In green are the heterozygous mutations in C3 described in this report and those described earlier by Frémeaux-Bacchi et al. (2008) that have functional consequences, in orange is the C3 mutation C3923ΔDG, the only mutation linked to dense deposit disease (DDD) that has been functionally characterized thus far (Martínez-Barricarte et al., 2010). (b) Enlarged image of the C3b region including the C3b_R592W_, C3b_R161W_ and C3b_I1157T_ mutations. (For interpretation of the references to color in this figure legend, the reader is referred to the web version of this article.)

C3b shows characteristic electron microscopy (EM) features that can be directly extrapolated to the atomic structure of C3b (Torreira et al., 2009, Nishida et al., 2006, Janssen et al., 2006). The most frequent view, by comparison to crystal structures, corresponds to a relatively compact conformation in which the TED domain is visualized attached to the MG ring. This conformation represents >90% of EM images in native C3b, likely maintained by charge interactions between the domains because increasing salt concentration above 50 mM NaCl resulted in dramatic conformational change with TED domain detachment from the MG ring (not shown). We have used these EM analyses to determine whether the overall structure of C3b is altered in the C3b_R592W_, C3b_R161W_ and C3b_I1157T_ mutants. C3b_R592W_ was indistinguishable from wt C3b with only a minor proportion of EM images (6% compared to 9% in wt C3b) presenting a detached TED domain (Fig. 6a). In contrast, EM analysis of mutants C3b_I1157T_ and C3b_R161W_ showed 68% and 36% respectively of EM images had TED displaced from MG domain. Considering that mutant C3 protein in the C3b_I1157T_ and C3b_R161W_ preparations represents approximately 50% of the C3b molecules, these data suggest that both I1157T and R161W mutations impact MG/TED domain interaction and overall C3b conformation, I1157T mutation having the strongest effect. These are important findings because a displaced TED has direct consequences for interaction of C3b with the regulators FH (Wu et al., 2009) and MCP (Persson et al., 2010) (Fig. 6, Fig. 7). We recently postulated that TED displacement contributes to the increased resistance of properdin-C3bBb complexes (Alcorlo et al., 2013) and iC3b (Alcorlo et al., 2011) to accelerated decay or inactivation by complement regulators.

The I1157T and R161W mutations localize to the TED and MG2 domains facing the MG1 and CUB domains respectively, and thus it is conceivable that these C3 mutations could affect, directly or indirectly, stability of contacts between the CUB-TED region and the MG ring. TED domain displacement in the C3b_I1157T_ and C3b_R161W_ mutants supports reduced stability of the contacts, which in turn provides a plausible explanation for their increased resistance to inactivation by MCP.

## 4 Discussion

aHUS is a rare, life-threatening renal pathology associated with complement dysregulation and endothelial cell injury, culminating in formation of microthrombi that occlude small vessels in kidney leading to severe renal impairment (Noris et al., 2009). aHUS has a strong genetic component (Pickering et al., 2007, Rodríguez de Córdoba et al., 2014) and is strongly associated with polymorphisms and mutations in activators (C3 and FB) and regulators (MCP, FI and FH) of the complement AP. We identified six carriers of C3 mutations (Fig. 1). All were heterozygotes and all, excepting a 10-year-old child in pedigree HUS107, have developed aHUS. None of these patients carried additional mutations in *CFH*, *MCP*, *CFI* or *CFB*, or *CFH-CFHR1/5* rearrangements. Disease severity varied considerably among the patients (Supplementary materials and methods). To investigate whether this clinical variability was influenced by other aHUS genetic risk factors, patients and family members were genotyped for *CFH* and *MCP* aHUS-associated polymorphisms (Caprioli et al., 2003, Esparza-Gordillo et al., 2005, Sánchez-Corral et al., 1989). Disease severity correlated with the presence of risk haplotypes in *CFH* and *MCP* in patients HUS19 and HUS107. Although the number of patients reported here is too small to draw definitive conclusions, the data suggest that the aHUS phenotype associated with the *C3* mutations is modulated by common polymorphisms conferring risk or protection from aHUS, as demonstrated before for mutations in other complement genes (Esparza-Gordillo et al., 2005, Esparza-Gordillo et al., 2006, Abarrategui-Garrido et al., 2009, Martinez-Barricarte et al., 2008). This observation is consistent with the recent demonstration that severity and penetrance of aHUS in C3 mutation carriers in the French cohort was influenced by the presence of the *MCP* risk haplotype (Roumenina et al., 2012), and that risk and protective *CFH* haplotypes impacted on risk in two large aHUS pedigrees (Sansbury et al., 2014).

MCP and FH are vital cofactors for FI-mediated inactivation of C3b by cleavage of the CUB domain. Previous studies of mutations in FH, MCP and C3 suggested that aHUS is caused by surface dysregulation of complement, while fluid phase regulatory capacity remains normal (Rodríguez de Córdoba et al., 2014). Data presented in this report support these suggestions. We illustrate here that despite our C3b mutants have reduced affinity for both FH and MCP, they show impaired regulatory function only for MCP. This is consistent with previous reports, testing several C3 mutations expressed as recombinant proteins, in which a decreased affinity for MCP and/or FH was observed by the majority of the mutant C3b protein tested. Importantly, the R161W mutant was included in those studies and when FI-cofactor activity by FH and MCP was tested in this mutant it was found that only the regulation by MCP was impaired (Roumenina, et al., 2012, Frémeaux-Bacchi et al., 2008, Schramm et al., 2015, Maga et al., 2010).

Alignment of MCP1-4 and FH1-4 structures onto C3b highlights important differences between these regulators regarding their interaction interfaces on C3b (Fig. 7a). SCR3 and SCR4 in both FH and MCP interact with overlapping areas of C3b spanning a region including MG2/CUB and MG1/TED domains; however, MCP SCR3-4 appears significantly more bent (Persson et al., 2010). Notably this region of C3b is the location of our aHUS-associated C3 mutations and the four C3 mutations previously described (Roumenina et al., 2012, Frémeaux-Bacchi et al., 2008), all with differential impact on FH and MCP (Fig. 7a). Unlike their homologues in FH, SCR1 and SCR2 in the MCP hockey stick shape are not in contact with C3b (Wu et al., 2009, Persson et al., 2010, Gordon et al., 1995). Consistent with these interactions, MCP SCR4 is particularly important for cofactor function (Persson et al., 2010), whereas FH cofactor activity is largely lost without SCR1, implying that the interaction of SCR1 and SCR2 with C3b is crucial for FH regulatory activities (Gordon et al., 1995). Overall, these data show that the location of a mutation in C3 dictates its impact on FH and MCP interaction with C3b and therefore on fluid phase or surface regulation. This provides an explanation for the distinction between the three aHUS-associated C3 mutations described here (R592W, R161W and I1157T) and the only functionally characterized dense deposit disease-associated C3 mutant (C3del923DG) (Martínez-Barricarte et al., 2010); they specifically impact the interactions between C3b and MCP or C3b and FH, respectively (Fig. 7).

A major novel finding from EM is that two of the mutations (R161W and I1157T) disrupt the overall structure of C3b by dislocating the TED domain from the MG ring, resulting in increased TED flexibility (Fig. 6). A structural model (Fig. 7b) illustrates that these mutations are close enough to contact regions between MG2/CUB and MG1/TED domains, respectively, to impair inter-domain interactions. This observation reinforces the concept that TED position is an important modulator of C3b interactions with complement regulators, as previously proposed for properdin-C3bBb complexes (Alcorlo et al., 2013) and iC3b (Alcorlo et al., 2011) to explain resistance to accelerated decay and inactivation by complement regulators. TED domain flexibility and dislocation away from the MG1–MG8 domain core has been shown for hydrolysed C3 (C3H2O) (Li et al., 2010), perhaps explaining differences between C3b and C3H2O in their interactions with complement regulators; for example, the affinity of FH for C3H2O is several fold weaker than the C3b–FH interaction (Heurich et al., 2011).

A recent report used analytical centrifugation, X-ray and neutron scattering to show TED domain detachment from MG1 domain when NaCl concentration was raised; a salt-bridge between residues E1031 in TED and R102 in MG1 domain was implicated in the interaction (Rodriguez et al., 2014). Our data are consistent with these results, EM images revealing conformational heterogeneity in positioning of the TED domain with respect to the MG ring and demonstrating that mutations around TED/MG contact regions dislocate the interaction resulting in increased TED flexibility and altered C3b conformation. The R592W mutation located in C3b MG6 does not disrupt TED location (Fig. 7); its functional impact thus involves a different mechanism, perhaps directly disrupting the C3b interaction surface thereby reducing FH and MCP affinity and MCP regulatory capacity.

In conclusion, we report functional, biochemical and structural explanations of the mechanism of pathogenicity of three aHUS-associated C3 mutations; all three mainly impair the regulatory action of MCP. These data further support the contention that aHUS is caused by surface-restricted AP dysregulation and illustrate the power of combining genetic, functional and structural studies of pathogenic mutations to explain mechanisms of disease.

## Disclosures

Authors declare no financial conflicts of interest. SRdeC has received honoraria from Alexion Pharmaceuticals for giving lectures and participating in advisory boards. None of these activities has had any influence on the results or interpretation in this article. BPM is a Consultant for Glaxo Smith Kline but receives no personal remuneration for this work. CLH has a contract of employment with GlaxoSmithKline; all work carried out in her lab on this project was completed prior to that employment.

## Figures and Tables

**Table 1:** Complement and genetic data in individuals carrying the *C3* mutations.

ID	C3 levels (80–177 mg/dL)	fB levels (>17 mg/dL)	C4 levels (14–47 mg/dL)	fH levels (5–35 mg/dL)	Additional aHUS genetic factors		
					*MCP*_GGAAC_	*CFH*_TGTGGT_	*CFH*_GATAAG_
HUS19	110	26.7	21.4	16.08	Yes (Hom)	No	No
HUS19 (I-2)	95	25.7	23.9	10.87	Yes (Het)	No	Yes (Het)
HUS19 (I-3)	107	25.7	15	19.19	Yes (Het)	No	Yes (Het)
HUS107	91.1	33.2	13.2	22.2	Yes (Het)	Yes (Hom)	No
H107 (III-3)*	92.5	22.8	16.5	15	No	Yes (Het)	Yes (Het)
HUS193	46.6	15.7	39.1	43.84	Yes (Het)	No	Yes (Het)
