# Supplementary material for: The molecular and structural bases for the association of complement C3 mutations with atypical hemolytic uremic syndrome
Source: Mol Immunol. 2015 Aug;66(2):263–73. doi: 10.1016/j.molimm.2015.03.248 (PMC4503813; doi:10.1016/j.molimm.2015.03.248)
Supplement: Supplementary file 1 [file mmc1.docx]

**SUPPLEMENTARY MATERIALS**

**Supplementary materials and methods**

**TFE Potein Digestion and MS/MS analysis**

2μg of sample were dried, resuspended in ammonium bicarbonate 100mM with 100%TFE denaturing agent and sonicated for 15 minutes. The reduction was carried out with 10mM DTT in 50mM ammonium bicarbonate, and the alkylation with 55 mM iodoacetamine in 50 mM ammonium bicarbonate. The samples were diluted 10-fold with 50 mM ammonium bicarbonate buffer.

Digestion was performed by the addition of trypsin (Promega, Fitchburg, WI) at a ratio of 1:10 (w/w) protease:protein at 37 °C overnight. The reaction was quenched 
by adding 1 µl TFA. The peptide mixtures from in-solution tryptic digestions were dissolved in 2% ACN and analyzed using nLC-MS/MS. The peptides were loaded onto a C18-A1 ASY-Column 2 cm, ID100 µm, 5µm precolumn (Proxeon, Thermo Scientific, Waltham, MA, USA) and then eluted with a linear gradient of 0–95% ACN in 0.1% aqueous solution of formic acid. The gradient was performed over 40 min by a ThermoEasy-nLC (Proxeon) at a flow-rate of 250 μL/min onto a NS-AC-11-dp3 Biosphere C18 capillary column, 75 µm, 16 cm, 3 µm (Nano Separations) to a stainless steel nano-bore emitter (Proxeon). The peptides were scanned and fragmented with an LTQ-OrbitrapVelos (ThermoScientific). Mass spectra files were searched using Sequest search engine through Thermo Proteome Discoverer 1.3.0.339.

**Supplementary Files**

**
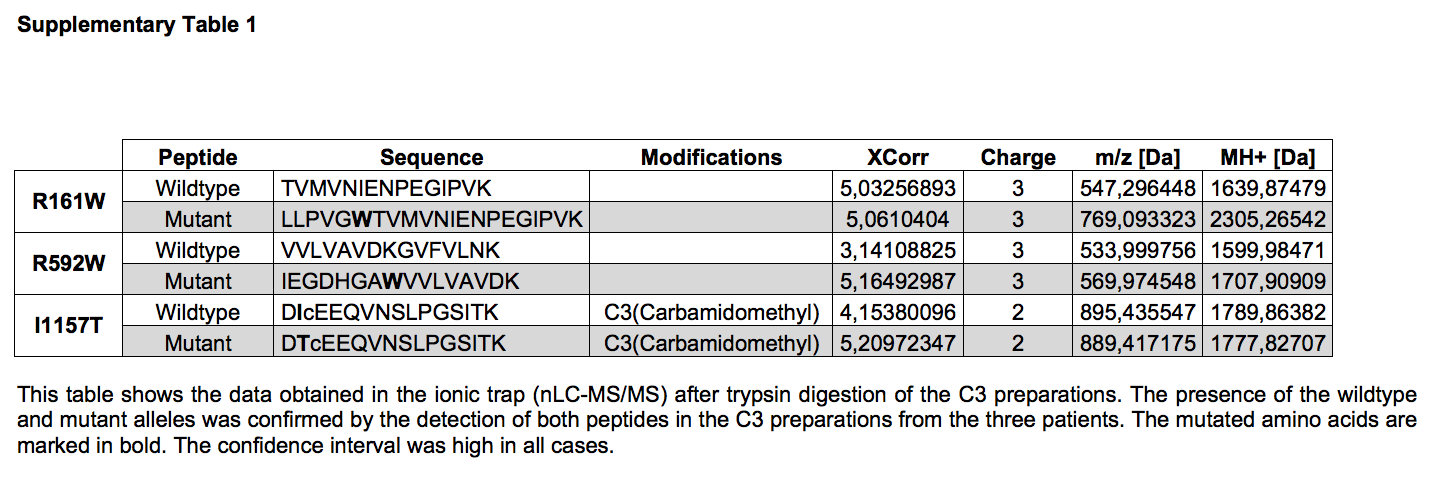
**

**Supplementary Figure 1.**
